# “Not All That Glitters Is Gold”: Paraspinal Cavernous Hemangioma Mimicking Paraganglioma on Somatostatin Receptor PET/CT

**DOI:** 10.3390/diagnostics15192456

**Published:** 2025-09-25

**Authors:** Cesare Michele Iacovitti, Ivan Cabrilo, Chiara Martinello, Marco Cuzzocrea, Gaetano Paone, Giorgio Treglia

**Affiliations:** 1 Division of Nuclear Medicine, Imaging Institute of Southern Switzerland, Ente Ospedaliero Cantonale, 6500 Bellinzona, Switzerland; cesaremichele.iacovitti@eoc.ch (C.M.I.); chiara.martinello@eoc.ch (C.M.); marco.cuzzocrea@eoc.ch (M.C.); gaetano.paone@eoc.ch (G.P.); 2 Division of Neurosurgery, Neurocenter of Southern Switzerland, Ente Ospedaliero Cantonale, 6900 Lugano, Switzerland; ivan.cabrilo@eoc.ch; 3 Faculty of Biomedical Sciences, Università della Svizzera Italiana, 6900 Lugano, Switzerland; 4 Faculty of Biology and Medicine, University of Lausanne, 1015 Lausanne, Switzerland

**Keywords:** somatostatin receptor, PET/CT, paraganglioma, cavernous hemangioma, hybrid imaging, nuclear medicine

## Abstract

We report the case of a 77-year-old male with a paraspinal mass at the Th11 level. Given its morphology and location, CT and MRI findings raised the suspicion of paraganglioma. A somatostatin receptor positron emission tomography/contrast-enhanced-CT (SSTR-PET/CE-CT) scan with Gallium-68 DOTATATE, performed for staging of suspected paraganglioma, demonstrated intense tracer uptake in the lesion, reinforcing the suspicion of a neuroendocrine tumor with increased SSTR expression. However, histopathology demonstrated a cavernous hemangioma with partial sclerosis, and immunohistochemistry showed strong endothelial SSTR expression. This case highlights the possibility of false-positive findings on SSTR PET/CT, as a cavernous hemangioma may closely mimic paraspinal paraganglioma in both imaging appearance and tracer uptake pattern. Notably, the intense tracer accumulation in this case was not solely related to the lesion’s hypervascularity but predominantly to the marked endothelial overexpression of SSTR demonstrated on immunohistochemistry.

**Figure 1:**
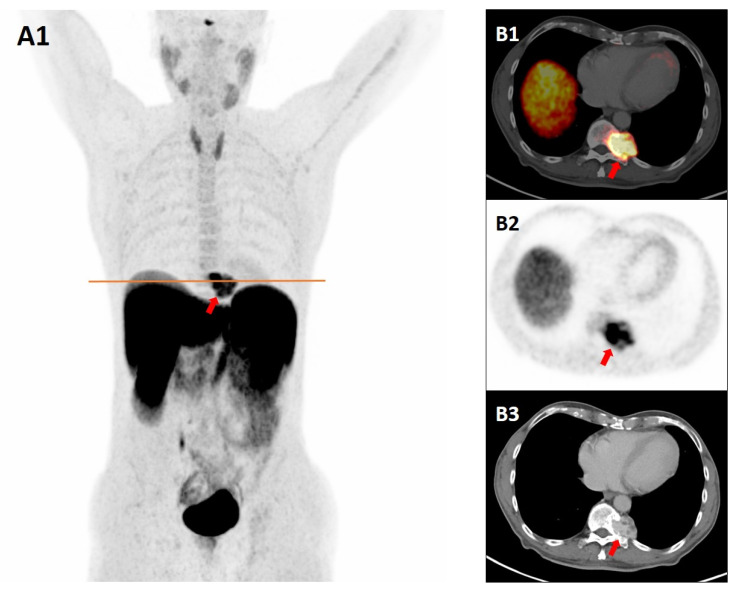
A 77-year-old male underwent computed tomography (CT) for evaluation of peripheral artery disease, which revealed a left paraspinal mass at the T11 level, adjacent to the costovertebral junction. The subsequent magnetic resonance imaging (MRI) revealed a 35 × 39 mm spinal/paraspinal lesion at the left Th11 level with homogeneous contrast enhancement, bone erosion, and sclerosis, suggestive of slow growth. Conventional imaging findings prompted consideration of both paraganglioma and a primary spinal tumor, particularly of chondroid origin, in the differential diagnosis. To assess for neuro-endocrine origin, the patient underwent plasma-free metanephrine screening and a somatostatin receptor positron emission tomography/contrast-enhanced-CT (SSTR-PET/CE-CT) scan with Gallium-68 DOTATATE (a radiolabeled somatostatin analog) for staging, given the well-established overexpression of somatostatin receptor subtype 2 (SSTR2) in neuroendocrine tumors [1,2,3]. Maximum intensity projection (MIP) PET image (**A1**), axial fused PET/CT (**B1**), PET (**B2**), and venous-phase CT image (**B3**) at the level of the orange line in A1 show the marked tracer accumulation corresponding to an expansile, osteoerosive, and hypervascular mass involving the left lateral margin of the Th11 vertebral body and pedicle (red arrows). Plasma-free metanephrine and catecholamine levels confirmed that the lesion was biochemically inactive, after which a CT-guided biopsy was considered safe and performed. Histopathology revealed a partially sclerotic, vascular lesion without atypia, consistent with cavernous hemangioma. Immunohistochemistry showed endothelial positivity for ERG, CD34, CD31, and SSTR2, and negativity for D2-40, chromogranin, synaptophysin, and INSM1, with Ki-67 < 5%. Strong endothelial SSTR2 expression accounted for the avid tracer uptake and the false-positive appearance mimicking paraganglioma on SSTR PET/CT. Although incidental hemangiomas localized in different anatomical sites have already been reported on SSTR-PET/CT with Gallium-68 DOTA-peptides [4,5,6,7,8,9,10,11], to our knowledge this is the first case documenting increased Gallium-68 DOTATATE uptake in a histologically confirmed cavernous hemangioma, a rare hemangioma subtype. According to a systematic review, incidental findings at SSTR-PET/CT mimicking neuroendocrine tumors are predominantly seen in the thyroid gland, spine, brain, and breast, and most of them are benign lesions [6]. Our case underscores the importance of considering cavernous hemangiomas in the differential diagnosis of SSTR-avid lesions, given their potential to mimic paragangliomas and other neuroendocrine tumors in both morphology and functional imaging characteristics.

## Data Availability

The raw data supporting the conclusions of this article will be made available by the authors on request.
